# Hospital Staffs and Fees at Inquests

**Published:** 1912-04-20

**Authors:** F. J. Waldo

**Affiliations:** H.M. Coroner for the City of London and Southwark.


### The Editor's Letter Box.

### HOSPITAL STAFFS AND FEES AT INQUESTS.

*To the Editor of* The Hospital.

Sir,—I would like to expand your short report of a recent inquest held by me at my City Court, at which a house-surgeon of St. Bartholomew's Hospital asked me to allow him a fee for giving evidence in a case dying in St. Bartholomew's, and in which I made an order for a *post-mortem* to be made by him. I told him if I gave a fee it would come out of my own pocket. Had the patient expired on the steps of the hospital before entry, he (the doctor) would be entitled to the statutory fee of £1 1s. for making the *post-mortem*, if on my order, and to £1 1s. for giving evidence. I told him I had done my best to alter an anomalous, an unequal, and, in my opinion, unfair state of the law of coroners. In every other court of law all legally qualified registered medical men were entitled and were paid fees for giving evidence. Such, however, is not the case in a coroner's court, and I advised him (the doctor) to get the City Members of Parliament—Mr. Balfour and Sir Frederick Banbury—and any other influential members, to take up the matter, and get an alteration of the law.

A Departmental Committee, which had sat some years and inquired into coroner's law, had recommended in their Report, published in 1910, in the following words (see p. 17): "Under s. 22 of the Coroners Act, 1887, where an inquest is held on the body of a person who has died in a county or other lunatic asylum, or any public hospital, infirmary, or other medical institution, whether the same be supported by endowment or by voluntary subscription, the medical officer whose duty it may have been to attend the deceased person as an officer of such institution, is not entitled to any fee or remuneration for attending to give evidence at the inquest or for making a *post-mortem* examination. Considerable doubt exists as to what institutions fall within this provision, and the practice of local authorities differs as to allowing or disallowing fees to officers of medical institutions."

"We have been unable to obtain from anyone who has appeared before us a defence of this provision on its merits. In the case, at any rate, of the London hospitals, not only the visiting staff, but also the house physicians and surgeons give their services to the hospital without remuneration. We fail to see why a man who gives his services out of charity should be penalised for so doing by being required to perform a public duty, wholly outside the objects of the charity, without receiving the fee paid to other persons performing the same public duty. In all other courts a medical witness gets costs according to the scale, irrespective of the fact that he may be attached to an institution of a charitable or quasi-charitable nature. We think that this inequitable provision in s. 22 of the Coroners Act, 1887, ought to be repealed, and, whatever may have been the original motives of policy for placing officers of medical institutions in a separate class and debarring them from receiving the fees payable to any other medical practitioner, we cannot see any reasons whatever at the present time for continuing to deprive them of fees."

I told the doctor that the Committee in making their suggestion that s. 22 should be repealed, had quoted my evidence given before them on February 26, 1909, which is as follows (see p. 73 of Report): Question 1900 (chairman, Mackenzie Chalmers). You can pay him (the doctor) a fee if he makes a *post-mortem* outside the hospital? A. Yes. Q. 1901. But you cannot pay him a fee if he makes it inside? A. No, I have no right to pay him any fee unless the patient is taken in dead. If the patient expires just outside the doors of the hospital, and I ordered a *post-mortem* and call upon a doctor in the hospital to make it, I pay him one guinea for the *post-mortem* and one guinea for giving evidence in my court. Q. 1902. Then it comes to this: that if the doctors in a hospital gratuitously give their services to patients during life, they are obliged to do gratuitous services after death? A. That is so; and the law is very doubtful, to my mind, on that point, as to whom the coroner has the right to pay and whom he has not, I mean in public institutions; there have been different rulings, as you know. Q. 1903. But the principle is, that if a doctor gives his services by way of charity, he has to do extra services which have nothing to do with his charitable work, for nothing? A. I believe that is the principle. I am not in agreement with it. Q. 1904. Do you know how that provision got into the Act? A. I have been told that it had to do with the body-snatchers, Burke and Hare. Q. 1905. But surely doctors' fees have nothing to do with body-snatching? A. They should not have anything to do with it. I think they ought all to be paid, every one of them. Q. 1906 (Dr. Willcox). Do the doctors at Poor-law infirmaries get paid? A. That is in the discretion of the county. I am under two jurisdictions. The City as regards disbursements is quite separate and apart from the London County Council, who pay my disbursements for Southwark. The ruling, I believe, of the London County Council is that in a workhouse and a workhouse infirmary you may pay the doctor; but I am a member of the Council of the Society of Coroners of England and Wales, and, of course, one hears things and gets the experience of other men in the country, and one hears of all kinds of things in different counties. The London County Council allows us to pay the doctor who makes the *post-mortem* examination and gives evidence from a workhouse or workhouse infirmary; but in other counties the coroners are not allowed to pay them. Q. 1907 (Chairman). It depends upon the County Council in each case? A. Yes. Q. 1908 (Dr. Willcox). Do you consider that fair? A. No, I do not; I always pay them myself. I do not think it is fair that they should not be paid. I should pay all or not any. In the City of London I may say I am given very much greater powers. Once appointed coroner, my signature is sufficient for most things. In the City I am under different conditions as regards disbursements; they allow me a great deal of latitude, which I think is right, in the public interest."

In answering the house-surgeon at the inquest in question, I told him and the jury that the Departmental Committee appointed by the late Home Secretary, Mr. (now Lord) Gladstone, had made many valuable and pressing suggestions other than that of the fair and proper payment of medical witnesses, such as the bestowal on a central authority (Lord Chancellor, Home Secretary, and a small advisory committee of coroners) of a power to make rules of practice and procedure; professional qualifications of coroners who, in future, are to be barristers, solicitors, or medical men; power to be given a coroner to appoint, provisionally, a second deputy in case of emergency, such as illness; strengthening of the law as regards the compulsory reporting of all deaths under anæsthetics to coroner, etc.; an alteration in the present bad method of calling together coroners' juries; the general appointment of trained police officials actually in the force as coroners' officers; and, what is, perhaps, the most needful of all reforms, in the words of the Committee: "We have come to the conclusion that the system of fire inquests established by the City of London Fire Inquests Act of 1888 (*re* non-fatal fires) has worked well in the City of London, and that the benefit of this system ought to be extended to the country at large." With this the Society of Coroners are in agreement. I may add that in my evidence before the Committee I advocated the adoption of the Australian practice of paying the pathologist a fee of two guineas (in place of the statutory British fee of one guinea) for making a *post-mortem* examination, and one guinea for giving evidence before the coroner.

I also suggested that in place of statutory fixed fees the more elastic method in force in courts other than that of the coroner, of leaving such matters with a Central Authority might with advantage be followed. The Committee in their Report recommended the adoption of both these suggestions. I do not agree with certain other of the recommendations of the Committee—e.g. *post-mortem* examination—without inquest, and abolition of view of the body by the jury. I see no prospect of legislation this session, much as it is to the public interest that the Government should draft a Bill and get it placed upon the Statute Book at the earliest possible period. Taken as a whole the recommendations in the Report of the Departmental Committee are, I think, distinctly favourable to the medical profession, and it would be well for those concerned if the profession would do all in their power to induce the Government to draft a Bill on the lines laid down in the Report of the Committee appointed by the Home Office.—Yours truly,

F. J. Waldo,

April 12. H.M. Coroner for the City of London and Southwark.

### OUT-PATIENTS AND SOLICITORS' TOUTS.

*To the Editor of* The Hospital.

